# jPOST environment accelerates the reuse and reanalysis of public proteome mass spectrometry data

**DOI:** 10.1093/nar/gkae1032

**Published:** 2024-11-11

**Authors:** Shujiro Okuda, Akiyasu C Yoshizawa, Daiki Kobayashi, Yushi Takahashi, Yu Watanabe, Yuki Moriya, Atsushi Hatano, Tomoyo Takami, Masaki Matsumoto, Norie Araki, Tsuyoshi Tabata, Mio Iwasaki, Naoyuki Sugiyama, Yoshio Kodera, Satoshi Tanaka, Susumu Goto, Shin Kawano, Yasushi Ishihama

**Affiliations:** Medical AI Center, Niigata University School of Medicine, 2−5274, Gakkocho-dori, Chuo-ku, Niigata 951-8514, Japan; Medical AI Center, Niigata University School of Medicine, 2−5274, Gakkocho-dori, Chuo-ku, Niigata 951-8514, Japan; Department of Omics and Systems Biology, Graduate School of Medical and Dental Sciences, Niigata University, 757 Ichibancho, Asahimachi-dori, Chuo-ku, Niigata 951-8510, Japan; Medical AI Center, Niigata University School of Medicine, 2−5274, Gakkocho-dori, Chuo-ku, Niigata 951-8514, Japan; Medical AI Center, Niigata University School of Medicine, 2−5274, Gakkocho-dori, Chuo-ku, Niigata 951-8514, Japan; Database Center for Life Science, Joint Support-Center for Data Science Research, Research Organization of Information and Systems, 178-4-4 Wakashiba, Kashiwa 277-0871, Japan; Department of Omics and Systems Biology, Graduate School of Medical and Dental Sciences, Niigata University, 757 Ichibancho, Asahimachi-dori, Chuo-ku, Niigata 951-8510, Japan; Department of Omics and Systems Biology, Graduate School of Medical and Dental Sciences, Niigata University, 757 Ichibancho, Asahimachi-dori, Chuo-ku, Niigata 951-8510, Japan; Department of Omics and Systems Biology, Graduate School of Medical and Dental Sciences, Niigata University, 757 Ichibancho, Asahimachi-dori, Chuo-ku, Niigata 951-8510, Japan; Department of Tumor Genetics and Biology, Graduate School of Medical Sciences, Faculty of Life Sciences, Kumamoto University, 1-1-1, Honjo, Chuo-ku, Kumamoto 860-0811, Japan; MassSoft, Sakyo-ku, Kyoto 606-8501, Japan; Department of Life Science Frontiers, Center for iPS Cell Research and Application, Kyoto University, Sakyo-ku, Kyoto 606-8507, Japan; Omics Research Center, National Cerebral and Cardiovascular Center, 6-1 Kishibe-Shimmachi, Suita, Osaka 564-8565, Japan; Graduate School of Pharmaceutical Sciences, Kyoto University, 46-29 Yoshidashimoadachi-cho, Sakyo-ku, Kyoto 606-8501, Japan; Center for Disease Proteomics, School of Science, Kitasato University, 1-15-1 Kitazato, Minami-ku, Sagamihara 252-0373, Japan; Trans-IT Co., Ltd., Mibu-machi, Tochigi 321-0204, Japan; Database Center for Life Science, Joint Support-Center for Data Science Research, Research Organization of Information and Systems, 178-4-4 Wakashiba, Kashiwa 277-0871, Japan; Database Center for Life Science, Joint Support-Center for Data Science Research, Research Organization of Information and Systems, 178-4-4 Wakashiba, Kashiwa 277-0871, Japan; School of Frontier Engineering, Kitasato University, 1-15-1 Kitazato, Minami-ku, Sagamihara 252-0373, Japan; Graduate School of Pharmaceutical Sciences, Kyoto University, 46-29 Yoshidashimoadachi-cho, Sakyo-ku, Kyoto 606-8501, Japan; Laboratory of Proteomics for Drug Discovery, National Institute of Biomedical Innovation, Health and Nutrition, Ibaraki, Osaka 567-0085, Japan

## Abstract

jPOST (https://jpostdb.org/) comprises jPOSTrepo (https://repository.jpostdb.org/) (over 2000 projects), a repository for proteome mass spectrometry data, the reanalysis of raw proteome data based on a standardised protocol using UniScore, and jPOSTdb (https://globe.jpostdb.org/) (over 600 datasets), a database that integrates the reanalysed data. The jPOST reanalysis protocol rescores MS/MS spectra using a new scale, UniScore, to evaluate the extent to which the spectral peaks correspond to the amino acid sequences identified by search engines. However, the metadata registered in the repository database is insufficient for conducting the reanalysis. To address this issue, the Japanese Proteomics Society launched a data journal, the Journal of Proteome Data and Methods (JPDM), which accepts data descriptor articles detailing metadata that can be reanalysed. Within jPOST, raw proteome data is reanalysed based on the metadata described in the JPDM data descriptor articles, utilising UniScore. The reanalysed data is deposited in jPOSTdb, and a link to the JPDM articles is added to jPOSTrepo. These reanalysis accelerations within the jPOST environment will promote FAIR data principles and open science.

## Graphical Abstract

**Graphical Abstract:**
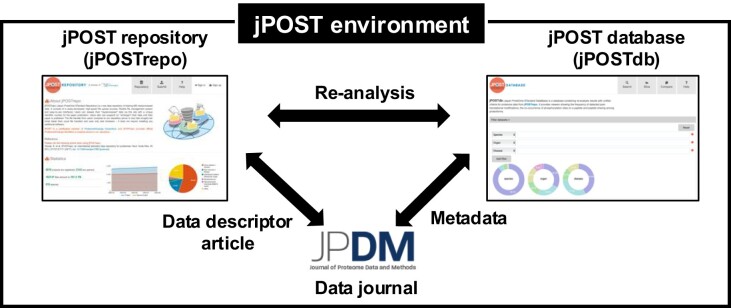

## Introduction

In recent years, research involving DNA and RNA using next-generation sequencing has become increasingly common, generating large amounts of data for researchers. Additionally, rapid advances in mass spectrometry have facilitated the acquisition of high-depth proteome data, leading to the widespread adoption of proteomics research within the scientific community. Moreover, other omics studies, including metabolomics and lipidomics, are now being conducted on a large scale, heralding an era in which many researchers manage extensive omics data.

Data obtained from various scientific research studies and the articles reporting these results must be widely accessible to the public and society. Based on this ‘open science’ concept (1), many datasets and articles have been published online in a format that can be accessed by anyone. In the context of open science, all data should be managed according to the FAIR Data Principles (2,3), which state that all data must be ‘Findable, Accessible, Interoperable and Reusable’. Various data repositories operate worldwide to implement open science and the FAIR data principles. Repository databases contain information on gene sequences (4–6), their expression (7,8), and other measurement data, allowing researchers globally to reference this information.

In 2015, we initiated the jPOST project to build an integrated proteome database by standardising, integrating, and managing different types of experimental proteome data from around the world based on FAIR data principles in proteomics (9). In 2016, we officially joined the ProteomeXchange Consortium (PXC) (10,11), and, as with other repository databases worldwide (12–15), jPOSTrepo (https://repository.jpostdb.org/) (9,16), a proteomics data repository compliant with international standards provided by the PXC, was launched in Japan. The PXC was selected as one of the Global Core Biodata Resources (GCBRs) in December 2022, a collection of 37 resources recognised by The Global Biodata Coalition (GBC) (https://globalbiodata.org/what-we-do/global-core-biodata-resources/) as critical to long-term funding and sustainability for life science and biomedical research worldwide. As a member of the PXC, jPOSTrepo has already accepted and managed a substantial amount of mass spectrometry (MS) data, and we have also developed and operated a database, ‘jPOSTdb’ (https://globe.jpostdb.org/) (17), which reanalyses these MS data using standardised protocols and integrates the results.

The reuse of publicly available data has become commonplace (18,19), with many researchers reusing and reanalysing data as part of new research efforts, in addition to confirming reproducibility. However, unlike next-generation sequencers, the reuse and reanalysis of raw MS data are affected by a variety of related information, much of which is not covered by the current metadata required for registration in repositories. To address this issue, the Japanese Proteomics Society launched a new data journal in 2019, the Journal of Proteome Data and Methods (JPDM) (https://www.jhupo.org/jpdm/) (20). The data descriptor, one of the JPDM article types, requires a more detailed description of the metadata of raw MS data in the repository, providing a level of detail that facilitates reanalysis. Such an initiative is unprecedented worldwide and could accelerate the reanalysis of data that is currently underutilised in repository databases.

This article summarises the major developments in the jPOST environment over the five years since the last NAR database update article (17) was published. Updates to jPOSTrepo, as well as the reanalysis and linkage with the database and JPDM, are reported.

## Current status of repository

**Figure 1.**
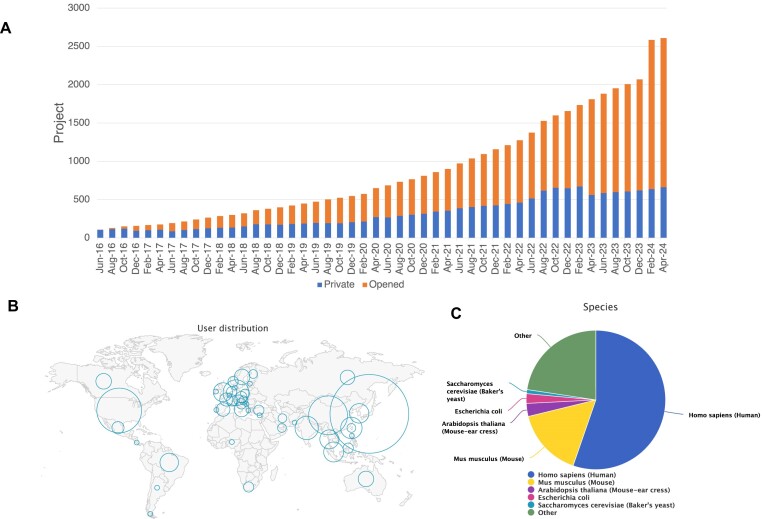
Statistics of submitted projects to the jPOSTrepo. (**A**) Number of projects submitted to the jPOSTrepo. The embargoed projects are shown in blue, and public projects are shown in orange. Note that the increase in the number of submissions at the beginning of 2024 is due to the reanalysis projects. (**B**) Global user distribution. (**C**) Species distribution.

Currently, 2876 projects are registered in jPOSTrepo (as of 30 August). Of these, 2143 projects are already publicly available, and their data can be reused worldwide (Figure 1A). In total, these data comprise more than 160 000 files and exceed 100 TB in size. In addition, many contributors are not from Asia, and data have been accepted from more than 50 countries worldwide (Figure 1B). The overwhelming majority of the species analysed in the submitted projects were humans, accounting for more than half of the data; however, more than 300 other species have been registered (Figure 1C).

## Reanalysis of MS data

The jPOST project has developed a standard protocol to reanalyse the raw MS data and has been actively conducting reanalyses based on this protocol. This reanalysis method rescored the MS/MS spectra using a new scale, named UniScore, to determine the extent to which the actual observed ions match the theoretical product ions of the peptide identified by the search engine (21). The parameters used are solely the number of matched b and y ions and the number of amino acid tags uniquely determined by being surrounded by these ions; therefore, it applies to any search engine. In the actual identification process, the results of several search engines are converted into UniScores and compared to select the best-matched peptide, and the false discovery rate (FDR) is also controlled by a target-decoy approach (22) using UniScores.

jPOSTrepo can accept proteome data acquired using various modalities, such as mass spectrometry data, including data-independent acquisition (DIA), parallel reaction monitoring (PRM), selected reaction monitoring (SRM), 2D electrophoresis data, and antibody data, not only in the complete submission format but also in the partial submission format. Currently, however, only data-dependent acquisition (DDA) mass spectrometry data are subject to reanalysis by jPOST, and the results are then integrated into the jPOSTdb. Data registration in repositories is primarily performed for submission to scientific journals, where various measurement data for a single article are often registered as a single project. However, in jPOST reanalysis, only MS raw files with identical metadata content are registered to constitute a single reanalysis ‘project’. Additionally, biological replication data, such as samples from different patients, are registered as ‘datasets’ within a single ‘project’. Currently, approximately 600 reanalysis datasets are registered in jPOSTdb.

When performing this reanalysis, the relationship between individual data files and their metadata is often unclear. Therefore, until now, the reanalysis was performed after scrutinising the articles associated with individual projects and manually collecting all of the relationships with the registered mass spectrometry raw data. It has been very difficult to increase the number of reanalyses in this way because the number of people who could perform this work is very limited, requiring knowledge of the experimental subject, proteomics methodology, data processing, and information about the repository.

## Data journal for reanalysis

In general, data reuse requires knowledge of how the data are processed and generated. Metadata describes this information, but for reasons such as the complexity of the input process, the metadata of datasets contained in many public databases is often inadequate for reuse and reanalysis. Therefore, data journals such as Scientific Data (23) and Data in Brief (24) publish articles in a category called Data Descriptor, which describes the data in detail. Following this trend, the Journal of Proteome Data and Methods (JPDM), led by the Japanese Proteome Society, was launched in September 2019. The journal also publishes articles in the data descriptor category, detailing how to acquire data for the proteome data repository. The objective of this study was to describe the data acquisition conditions in detail, including the correspondence between raw data files and metadata, so that anyone can reanalyse the data. Additionally, a Digital Object Identifier (DOI) is assigned to articles published in the JPDM. Data descriptor articles, which are the main content of the JPDM, are currently limited to raw proteomic data obtained using mass spectrometry, but any public data registered in any repository other than jPOST can be submitted. Currently, jPOST conducts reanalyses based on these data descriptor articles and integrates the reanalysed data.

The JPDM data descriptor article submissions include a dedicated Microsoft Excel file describing the detailed metadata. This Excel file is formatted according to the Sample and Data Relationship Format for Proteomics (SDRF-Proteomics) (25,26), which allows the automatic collection of metadata at a level that can be reanalysed. There are several groups for inputting metadata, and each group corresponds to the four presets of sample/fractionation/enzyme and modification/MS modes in jPOSTrepo. To further emphasise the analysis process, a separate group called ‘Software Setting’ has been created.

**Figure 2.**
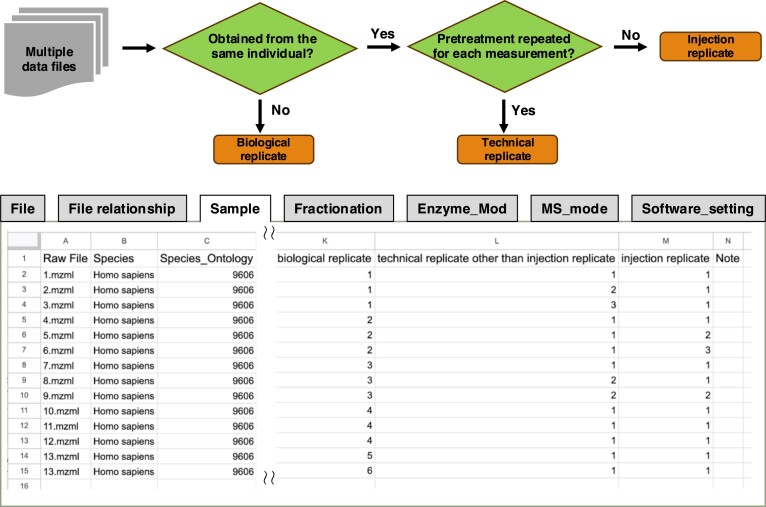
Classification criteria for replicates and an example of the metadata description from the JPDM Data Descriptor article. Biological replicates are classified as separate datasets within the same project, while injection replicates are processed by merging the results of the Peptide-Spectrum Match (PSM).

The contents of each raw data file were described to meet the requirements of SDRF-Proteomics, and the contents of each item were organised and enhanced for easy reuse, especially for samples. Specifically, sample attributes are classified not only by ‘Organ’, with humans and mice in mind, but also by ‘Tissue’, considering other species and by ‘Cell Line’ and ‘Disease’ in a hierarchical classification to prepare for future reuse. Additionally, a pre-processing field is provided for input when the content of a file cannot be distinguished by any of the entries. Three types of replicates were prepared to describe the corresponding relationships: biological replicates, which are used for samples such as those from patients; technical replicates, which correspond to cases where multiple vials containing the same sample are subjected to the same treatment; and injection replicates, which correspond to cases where a sample in a single vial is assayed multiple times (Figure 2).

Data descriptor articles are reviewed by multiple peer reviewers, as are regular research papers, subject to the condition that they do not describe scientific conclusions, and are required to present precise information that is necessary for data reuse.

## Collaboration with JPDM

**Figure 3.**
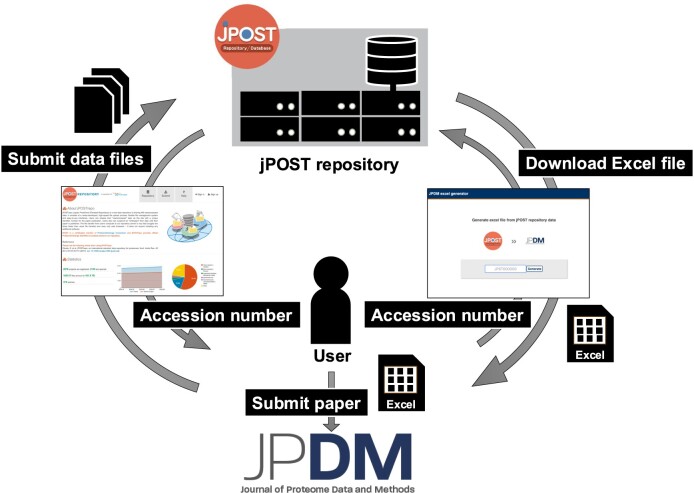
Collaboration between jPOST and JPDM.

In the case of a project registered in jPOSTrepo, a function automatically provides a file with the relevant items of this Excel file filled in based on the information in the metadata already registered in the repository. In addition to the site (https://repository.jpostdb.org/jpdm-excel/) where the Excel file can be obtained by entering the project ID, a link to the JPDM Excel file output was added to the project information table on each user's MyPage. Therefore, for the projects already registered in jPOSTrepo, it is relatively easy to prepare an Excel file for this metadata description, as additional information such as replicates need only be entered into the output Excel file. Furthermore, when a JPDM Data Descriptor article is accepted and published, a link to the article is automatically added to the jPOST project, providing a function to accelerate data reuse and reanalysis through mutual collaboration between jPOSTrepo and JPDM (Figure 3). Generally, when a researcher reanalyses the proteome data, only the PXD IDs are cited. However, if a researcher publishes a JPDM data descriptor, they can also be cited. This is important for researchers who generate and publish proteome data in terms of the number of citations of their work.

## Discussion

Repository databases are required to manage research data sustainably entrusted to researchers worldwide and to contribute to future scientific research by confirming the reproducibility of research and reanalysing the data. Although entrusted data should be managed based on the FAIR Data Principles, the current MS repository database is not fully fulfilling its role in terms of ‘Reuse.’ This is because the reanalysis of MS data requires the division of datasets into reanalysable units, such as experimental setup, preprocessing information, replicates, and so on. This is quite different from division by oligo sequences in next-generation sequencers, which is a complex mechanism. Therefore, obtaining more detailed metadata to enable reanalysis is crucial.

In collaboration with the academic community, we proposed a methodology to compensate for this problem in the form of a data descriptor article. We have shown that the metadata submitted in the Data Descriptor is sufficient for typical experiments; however, there is still the possibility that they may not be sufficient for more complex conditions. Despite these limitations, repository databases are obligated to provide sustainability in accordance with the FAIR principles for data; thus, it is necessary to continue to explore measures to further accelerate reanalysis.

## Data Availability

The resources can be accessed through the jPOST home page at https://jpostdb.org/. Data stored in jPOSTrepo are available at ftp://ftp.jpostdb.org/ and all the data are linked from each project website.

## References

[1] Craig R. , Cortens J.P., Beavis R.C. Open source system for analyzing, validating, and storing protein identification data. J. Proteome Res. 2004; 3:1234–1242. 15595733 10.1021/pr049882h

[2] Editorial FAIR principles for data stewardship. Nat. Genet. 2016; 48:343–343. 27023771 10.1038/ng.3544

[3] Wilkinson M.D. , Dumontier M., Aalbersberg I..J., Appleton G., Axton M., Baak A., Blomberg N., Boiten J.W., da Silva Santos L.B., Bourne P.E. et al. The FAIR Guiding Principles for scientific data management and stewardship. Sci. Data. 2016; 3:160018. 26978244 10.1038/sdata.2016.18 PMC4792175

[4] Clark K. , Karsch-Mizrachi I., Lipman D.J., Ostell J., Sayers E.W. GenBank. Nucleic Acids Res. 2016; 44:D67–D72. 26590407 10.1093/nar/gkv1276 PMC4702903

[5] Kodama Y. , Mashima J., Kosuge T., Ogasawara O. DDBJ update: the Genomic Expression Archive (GEA) for functional genomics data. Nucleic Acids Res. 2019; 47:D69–D73. 30357349 10.1093/nar/gky1002 PMC6323915

[6] Leinonen R. , Nardone F., Oyewole O., Redaschi N., Stoehr P. The EMBL sequence version archive. Bioinformatics. 2003; 19:1861–1862. 14512364 10.1093/bioinformatics/btg248

[7] Papatheodorou I. , Fonseca N.A., Keays M., Tang Y.A., Barrera E., Bazant W., Burke M., Füllgrabe A., Fuentes A.M.P., George N. et al. Expression Atlas: gene and protein expression across multiple studies and organisms. Nucleic Acids Res. 2018; 46:D246–D251. 29165655 10.1093/nar/gkx1158 PMC5753389

[8] Barrett T. , Wilhite S.E., Ledoux P., Evangelista C., Kim I.F., Tomashevsky M., Marshall K.A., Phillippy K.H., Sherman P.M., Holko M. et al. NCBI GEO: archive for functional genomics data sets–update. Nucleic Acids Res. 2013; 41:D991–D995. 23193258 10.1093/nar/gks1193 PMC3531084

[9] Okuda S. , Watanabe Y., Moriya Y., Kawano S., Yamamoto T., Matsumoto M., Takami T., Kobayashi D., Araki N., Yoshizawa A.C. et al. JPOSTrepo: an international standard data repository for proteomes. Nucleic Acids Res. 2017; 45:D1107–D1111. 27899654 10.1093/nar/gkw1080 PMC5210561

[10] Deutsch E.W. , Bandeira N., Perez-Riverol Y., Sharma V., Carver J.J., Mendoza L., Kundu D.J., Wang S., Bandla C., Kamatchinathan S. et al. The ProteomeXchange consortium at 10 years: 2023 update. Nucleic Acids Res. 2023; 51:D1539–D1548. 36370099 10.1093/nar/gkac1040 PMC9825490

[11] Vizcaíno J.A. , Deutsch E.W., Wang R., Csordas A., Reisinger F., Ríos D., Dianes J.A., Sun Z., Farrah T., Bandeira N. et al. ProteomeXchange provides globally coordinated proteomics data submission and dissemination. Nat. Biotechnol. 2014; 32:223–226. 24727771 10.1038/nbt.2839 PMC3986813

[12] Vizcaíno J.A. , Csordas A., Del-Toro N., Dianes J.A., Griss J., Lavidas I., Mayer G., Perez-Riverol Y., Reisinger F., Ternent T. et al. 2016 update of the PRIDE database and its related tools. Nucleic Acids Res. 2016; 44:D447–D456. 26527722 10.1093/nar/gkv1145 PMC4702828

[13] Farrah T. , Deutsch E.W., Kreisberg R., Sun Z., Campbell D.S., Mendoza L., Kusebauch U., Brusniak M.-Y., Hüttenhain R., Schiess R. et al. PASSEL: the PeptideAtlas SRMexperiment library. Proteomics. 2012; 12:1170–1175. 22318887 10.1002/pmic.201100515 PMC3832291

[14] Sharma V. , Eckels J., Schilling B., Ludwig C., Jaffe J.D., MacCoss M.J., MacLean B. Panorama public: a public repository for quantitative data sets processed in skyline. Mol. Cell. Proteomics. 2018; 17:1239–1244. 29487113 10.1074/mcp.RA117.000543 PMC5986241

[15] Ma J. , Chen T., Wu S., Yang C., Bai M., Shu K., Li K., Zhang G., Jin Z., He F. et al. Iprox: an integrated proteome resource. Nucleic Acids Res. 2019; 47:D1211–D1217. 30252093 10.1093/nar/gky869 PMC6323926

[16] Watanabe Y. , Yoshizawa A.C., Ishihama Y., Okuda S. The jPOST Repository as a Public Data Repository for Shotgun Proteomics. Methods Mol. Biol. 2021; 2259:309–322. 33687724 10.1007/978-1-0716-1178-4_20

[17] Moriya Y. , Kawano S., Okuda S., Watanabe Y., Matsumoto M., Takami T., Kobayashi D., Yamanouchi Y., Araki N., Yoshizawa A.C. et al. The jpost environment: an integrated proteomics data repository and database. Nucleic Acids Res. 2019; 47:D1218–D1224. 30295851 10.1093/nar/gky899 PMC6324006

[18] Dai C. , Pfeuffer J., Wang H., Zheng P., Käll L., Sachsenberg T., Demichev V., Bai M., Kohlbacher O., Perez-Riverol Y. quantms: a cloud-based pipeline for quantitative proteomics enables the reanalysis of public proteomics data. Nat. Methods. 2024; 21:1603–1607. 38965444 10.1038/s41592-024-02343-1 PMC11399091

[19] Drew K. , Lee C., Huizar R.L., Tu F., Borgeson B., McWhite C.D., Ma Y., Wallingford J.B., Marcotte E.M. Integration of over 9, 000 mass spectrometry experiments builds a global map of human protein complexes. Mol. Syst. Biol. 2017; 13:932. 28596423 10.15252/msb.20167490 PMC5488662

[20] Ishihama Y. From bench to Internet: sharing proteomics data and methods through the Open Access Journal. J. Proteome Data Methods. 2019; 1:1.

[21] Tabata T. , Yoshizawa A.C., Ogata K., Chang C.-H., Araki N., Sugiyama N., Ishihama Y. UniScore, a unified and universal measure for peptide identification by multiple search engines. 2024; bioRxiv doi:13 October 2024, preprint: not peer reviewed 10.1101/2024.10.09.617445. PMC12272588 40466863

[22] Elias J.E. , Gygi S.P. Target-decoy search strategy for mass spectrometry-based proteomics. Methods Mol. Biol. 2010; 604:55–71. 20013364 10.1007/978-1-60761-444-9_5 PMC2922680

[23] Editorial More bang for your byte. Sci. Data. 2014; 1:140010. 25977768 10.1038/sdata.2014.10 PMC4322585

[24] Wang H.R. ‘Publish or perish’: should this still be true for your data?. Data Brief. 2014; 1:85–86. 26217694 10.1016/j.dib.2014.11.005 PMC4459564

[25] Deutsch E.W. , Bandeira N., Sharma V., Perez-Riverol Y., Carver J.J., Kundu D.J., García-Seisdedos D., Jarnuczak A.F., Hewapathirana S., Pullman B.S. et al. The ProteomeXchange consortium in 2020: enabling ‘big data’ approaches in proteomics. Nucleic Acids Res. 2020; 48:D1145–D1152. 31686107 10.1093/nar/gkz984 PMC7145525

[26] Dai C. , Füllgrabe A., Pfeuffer J., Solovyeva E.M., Deng J., Moreno P., Kamatchinathan S., Kundu D.J., George N., Fexova S. et al. A proteomics sample metadata representation for multiomics integration and big data analysis. Nat. Commun. 2021; 12:5854. 34615866 10.1038/s41467-021-26111-3 PMC8494749

